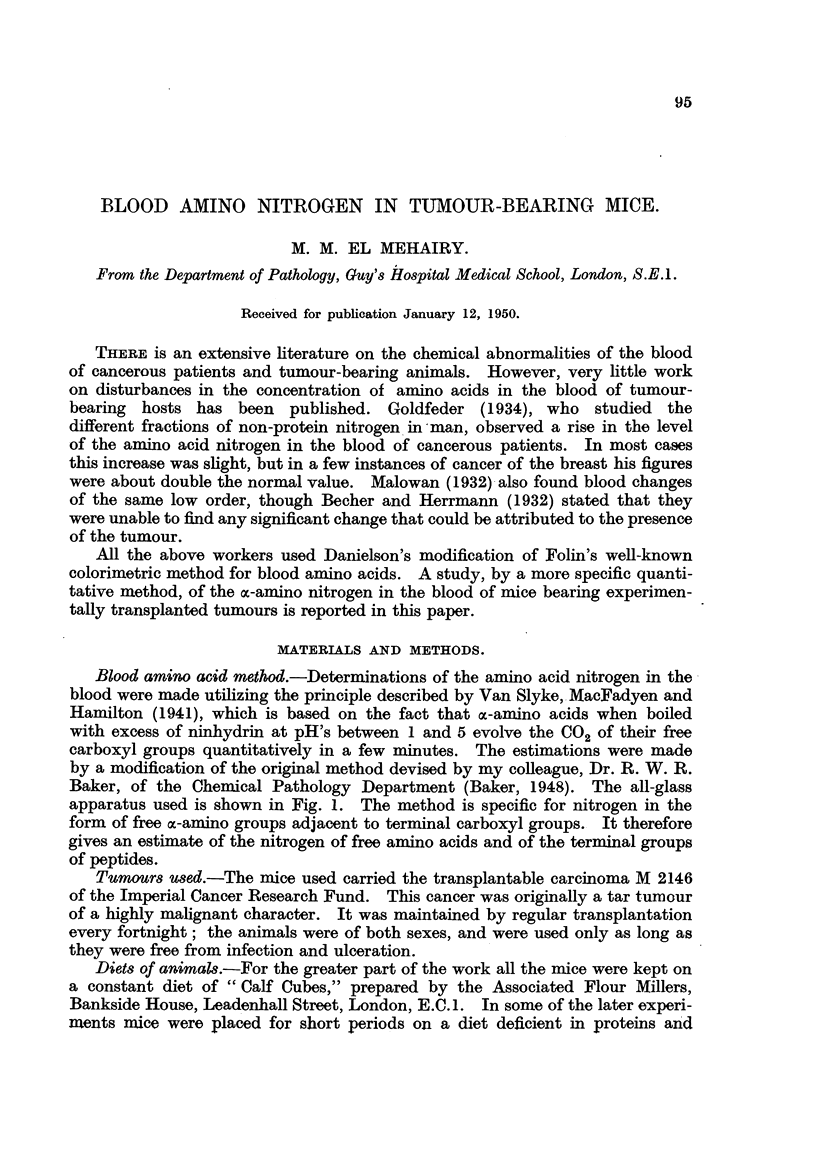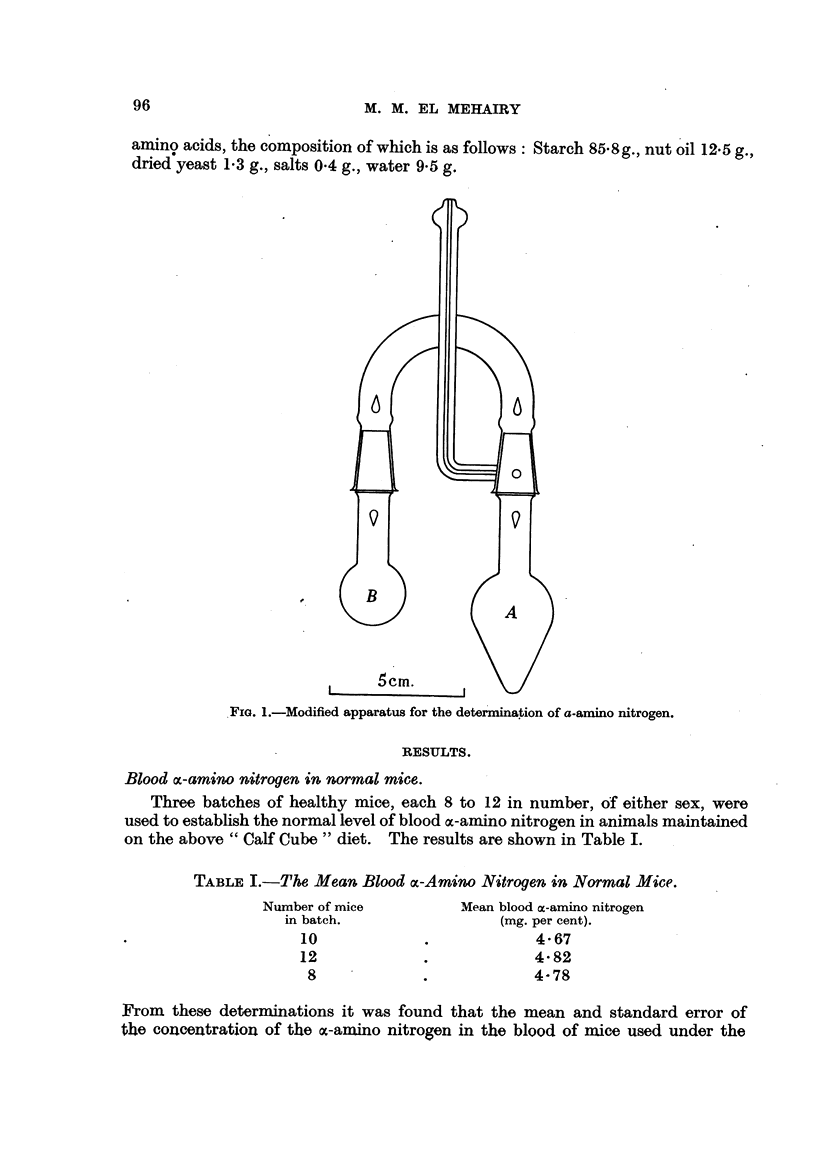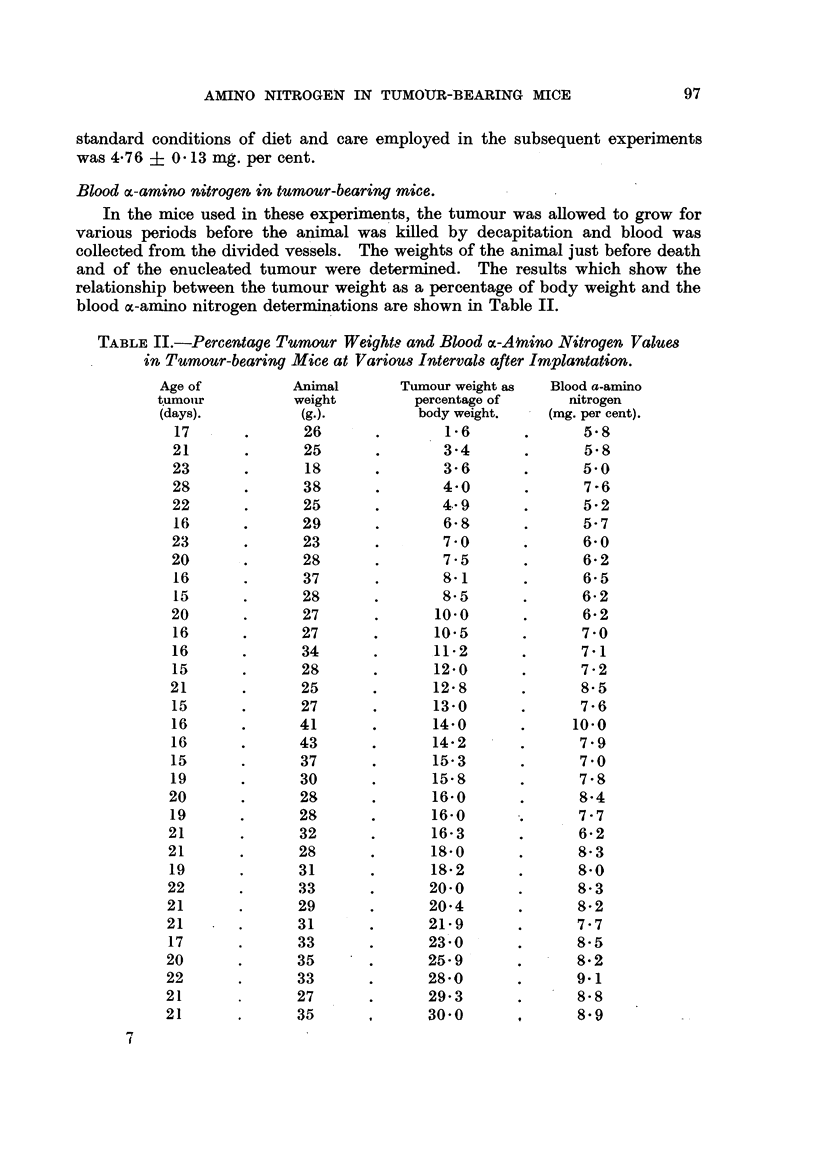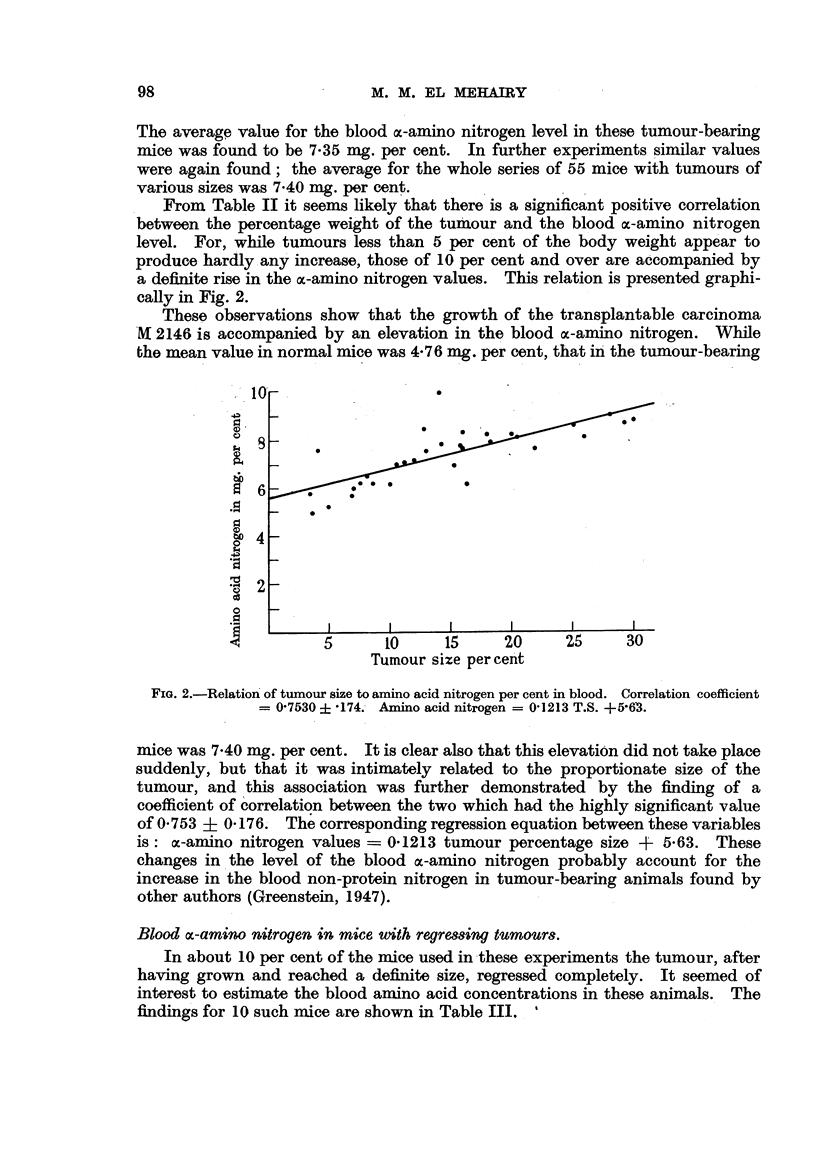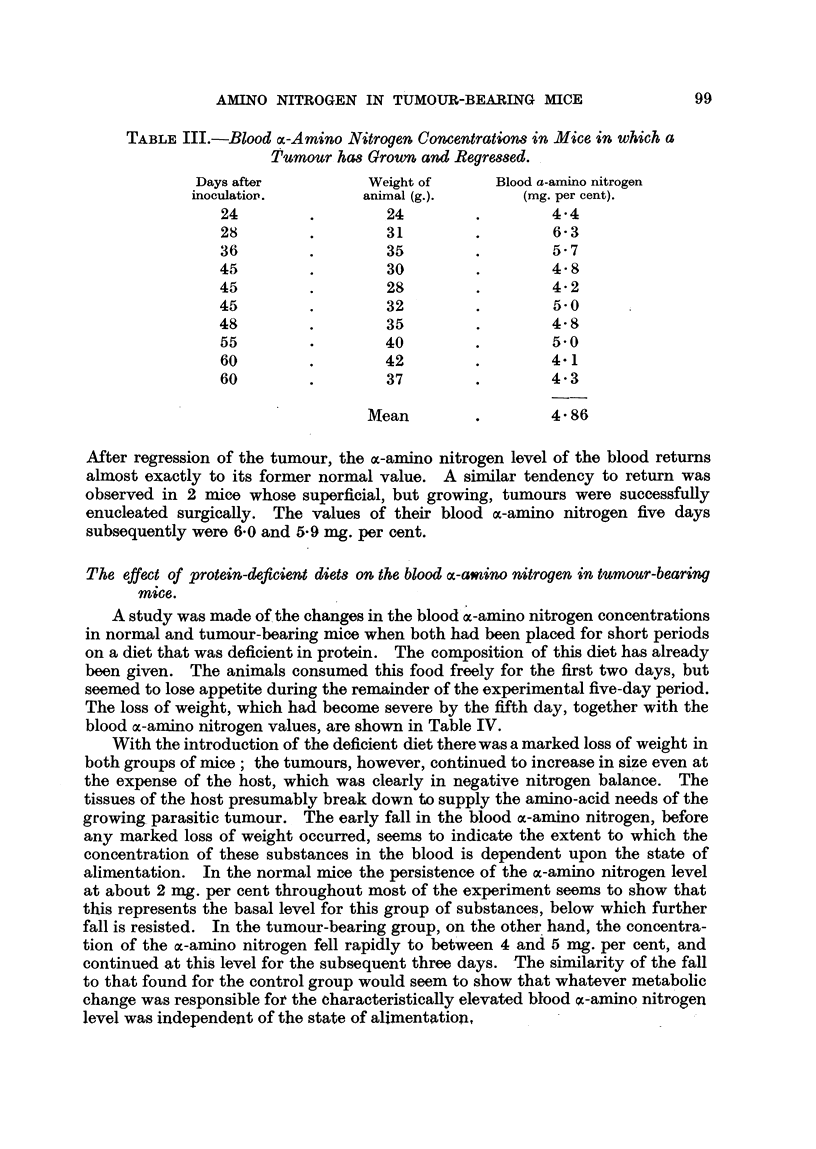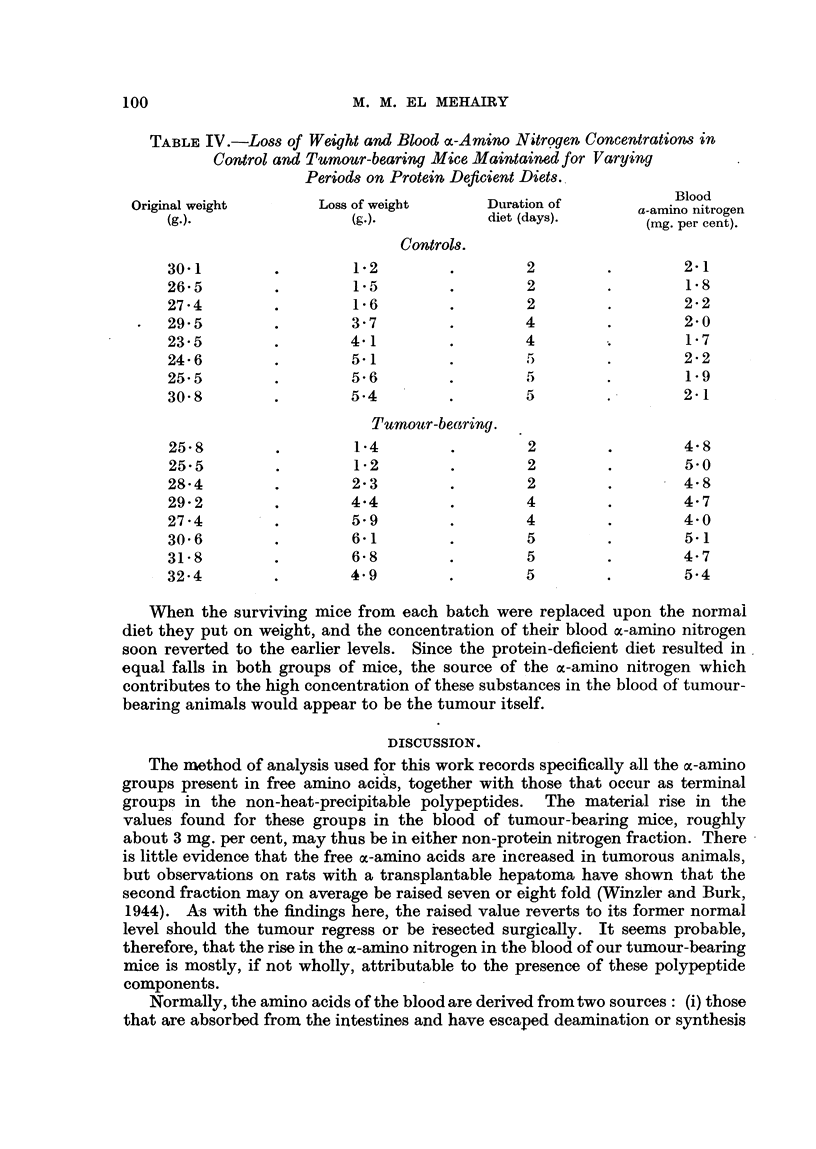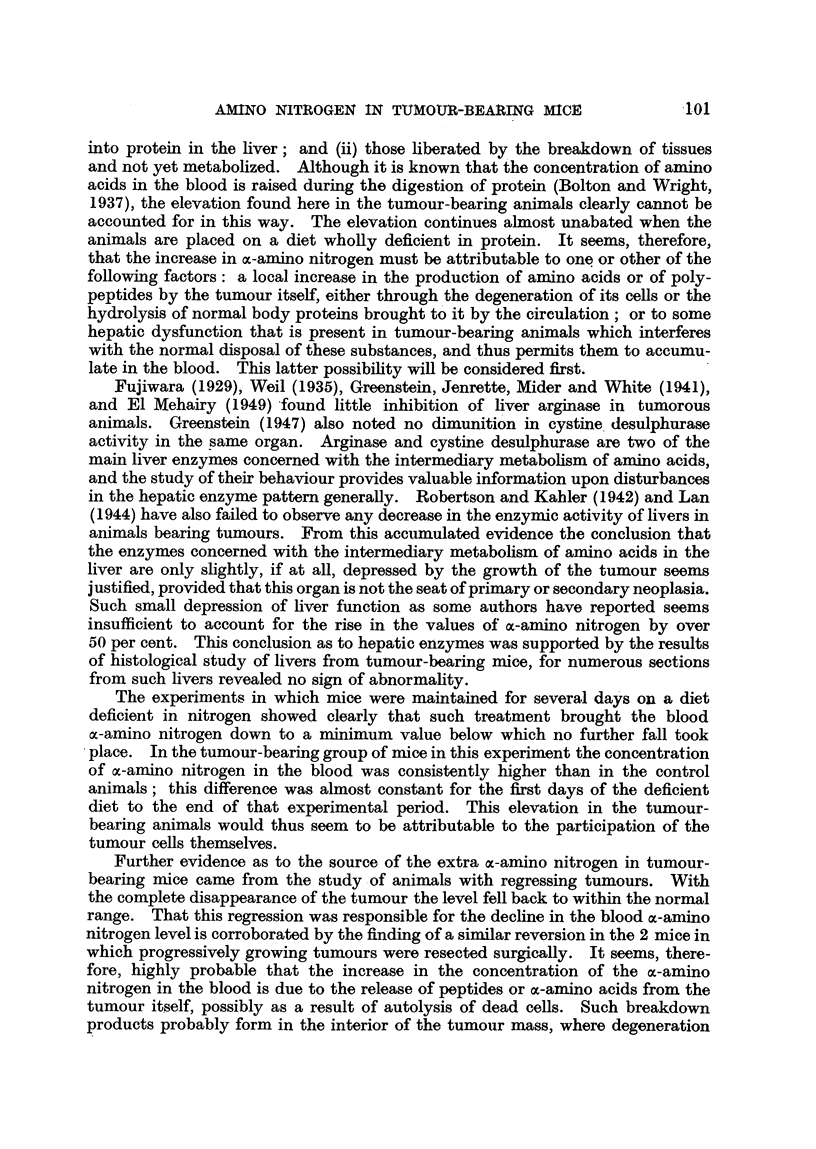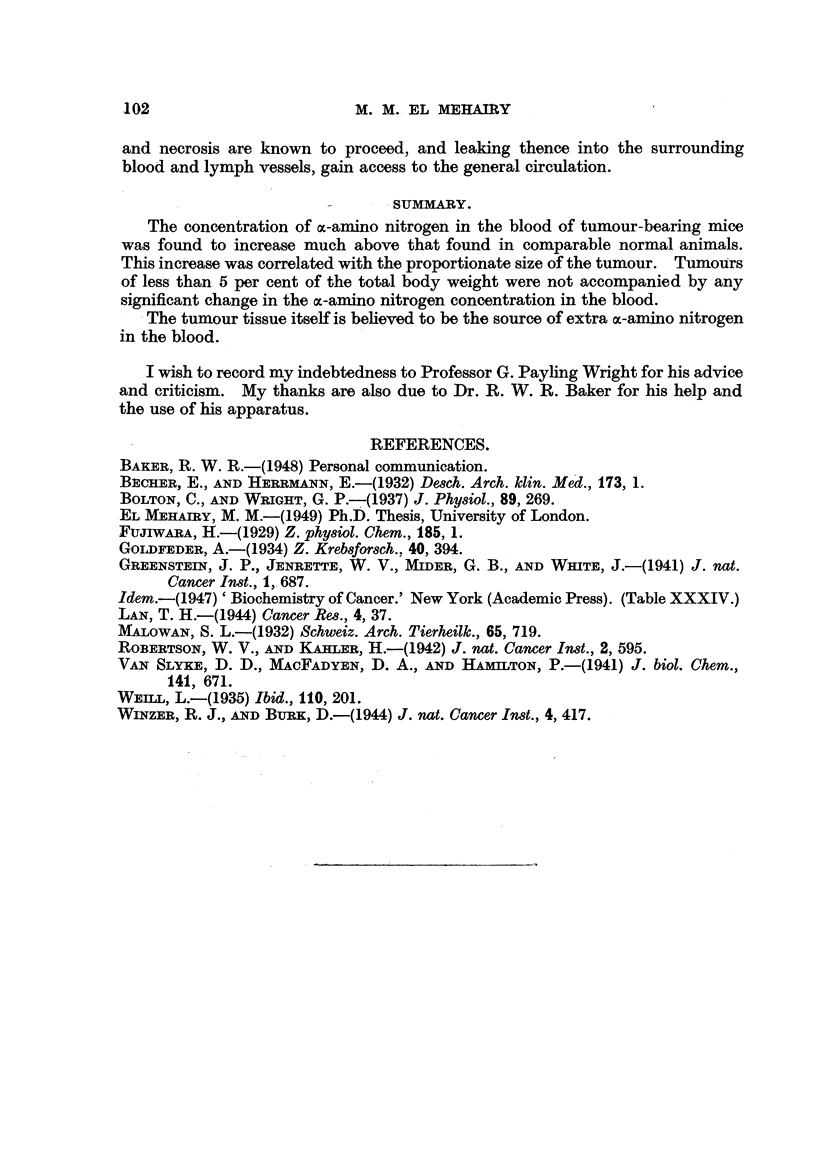# Blood Amino Nitrogen in Tumour-bearing Mice

**DOI:** 10.1038/bjc.1950.8

**Published:** 1950-03

**Authors:** M. M. El Mehairy


### BLOOD AMINO NITROGEN IN TUMOUR-BEARING MICE.

M. M. EL MEHAIRY.

*From the Department of Pathology, Guy's Hospital Medical School, London, S.E.1.*

Received for publication January 12, 1950.

THERE is an extensive literature on the chemical abnormalities of the blood of cancerous patients and tumour-bearing animals. However, very little work on disturbances in the concentration of amino acids in the blood of tumour-bearing hosts has been published. Goldfeder (1934), who studied the different fractions of non-protein nitrogen in man, observed a rise in the level of the amino acid nitrogen in the blood of cancerous patients. In most cases this increase was slight, but in a few instances of cancer of the breast his figures were about double the normal value. Malowan (1932) also found blood changes of the same low order, though Becher and Herrmann (1932) stated that they were unable to find any significant change that could be attributed to the presence of the tumour.

All the above workers used Danielson's modification of Folin's well-known colorimetric method for blood amino acids. A study, by a more specific quantitative method, of the α-amino nitrogen in the blood of mice bearing experimentally transplanted tumours is reported in this paper.

### MATERIALS AND METHODS.

*Blood amino acid method.*—Determinations of the amino acid nitrogen in the blood were made utilizing the principle described by Van Slyke, MacFadyen and Hamilton (1941), which is based on the fact that α-amino acids when boiled with excess of ninhydrin at pH's between 1 and 5 evolve the $CO_2$ of their free carboxyl groups quantitatively in a few minutes. The estimations were made by a modification of the original method devised by my colleague, Dr. R. W. R. Baker, of the Chemical Pathology Department (Baker, 1948). The all-glass apparatus used is shown in Fig. 1. The method is specific for nitrogen in the form of free α-amino groups adjacent to terminal carboxyl groups. It therefore gives an estimate of the nitrogen of free amino acids and of the terminal groups of peptides.

*Tumours used.*—The mice used carried the transplantable carcinoma M 2146 of the Imperial Cancer Research Fund. This cancer was originally a tar tumour of a highly malignant character. It was maintained by regular transplantation every fortnight; the animals were of both sexes, and were used only as long as they were free from infection and ulceration.

*Diets of animals.*—For the greater part of the work all the mice were kept on a constant diet of "Calf Cubes," prepared by the Associated Flour Millers, Bankside House, Leadenhall Street, London, E.C.1. In some of the later experiments mice were placed for short periods on a diet deficient in proteins and amino acids, the composition of which is as follows : Starch 85·8 g., nut oil 12·5 g., dried yeast 1·3 g., salts 0·4 g., water 9·5 g.

FIG. 1.—Modified apparatus for the determination of *a*-amino nitrogen.

### RESULTS.

### *Blood α-amino nitrogen in normal mice.*

Three batches of healthy mice, each 8 to 12 in number, of either sex, were used to establish the normal level of blood α-amino nitrogen in animals maintained on the above "Calf Cube" diet. The results are shown in Table I.

TABLE I.—*The Mean Blood α-Amino Nitrogen in Normal Mice.*

| Number of mice in batch. | Mean blood α-amino nitrogen (mg. per cent). |
|---|---|
| 10 | 4·67 |
| 12 | 4·82 |
| 8 | 4·78 |

From these determinations it was found that the mean and standard error of the concentration of the α-amino nitrogen in the blood of mice used under the standard conditions of diet and care employed in the subsequent experiments was $4{\cdot}76 \pm 0{\cdot}13$ mg. per cent.

*Blood α-amino nitrogen in tumour-bearing mice.*

In the mice used in these experiments, the tumour was allowed to grow for various periods before the animal was killed by decapitation and blood was collected from the divided vessels. The weights of the animal just before death and of the enucleated tumour were determined. The results which show the relationship between the tumour weight as a percentage of body weight and the blood α-amino nitrogen determinations are shown in Table II.

TABLE II.—*Percentage Tumour Weights and Blood α-Amino Nitrogen Values in Tumour-bearing Mice at Various Intervals after Implantation.*

| Age of tumour (days). | Animal weight (g.). | Tumour weight as percentage of body weight. | Blood α-amino nitrogen (mg. per cent). |
|---|---|---|---|
| 17 | 26 | 1·6 | 5·8 |
| 21 | 25 | 3·4 | 5·8 |
| 23 | 18 | 3·6 | 5·0 |
| 28 | 38 | 4·0 | 7·6 |
| 22 | 25 | 4·9 | 5·2 |
| 16 | 29 | 6·8 | 5·7 |
| 23 | 23 | 7·0 | 6·0 |
| 20 | 28 | 7·5 | 6·2 |
| 16 | 37 | 8·1 | 6·5 |
| 15 | 28 | 8·5 | 6·2 |
| 20 | 27 | 10·0 | 6·2 |
| 16 | 27 | 10·5 | 7·0 |
| 16 | 34 | 11·2 | 7·1 |
| 15 | 28 | 12·0 | 7·2 |
| 21 | 25 | 12·8 | 8·5 |
| 15 | 27 | 13·0 | 7·6 |
| 16 | 41 | 14·0 | 10·0 |
| 16 | 43 | 14·2 | 7·9 |
| 15 | 37 | 15·3 | 7·0 |
| 19 | 30 | 15·8 | 7·8 |
| 20 | 28 | 16·0 | 8·4 |
| 19 | 28 | 16·0 | 7·7 |
| 21 | 32 | 16·3 | 6·2 |
| 21 | 28 | 18·0 | 8·3 |
| 19 | 31 | 18·2 | 8·0 |
| 22 | 33 | 20·0 | 8·3 |
| 21 | 29 | 20·4 | 8·2 |
| 21 | 31 | 21·9 | 7·7 |
| 17 | 33 | 23·0 | 8·5 |
| 20 | 35 | 25·9 | 8·2 |
| 22 | 33 | 28·0 | 9·1 |
| 21 | 27 | 29·3 | 8·8 |
| 21 | 35 | 30·0 | 8·9 |

The average value for the blood α-amino nitrogen level in these tumour-bearing mice was found to be 7·35 mg. per cent. In further experiments similar values were again found; the average for the whole series of 55 mice with tumours of various sizes was 7·40 mg. per cent.

From Table II it seems likely that there is a significant positive correlation between the percentage weight of the tumour and the blood α-amino nitrogen level. For, while tumours less than 5 per cent of the body weight appear to produce hardly any increase, those of 10 per cent and over are accompanied by a definite rise in the α-amino nitrogen values. This relation is presented graphically in Fig. 2.

These observations show that the growth of the transplantable carcinoma M 2146 is accompanied by an elevation in the blood α-amino nitrogen. While the mean value in normal mice was 4·76 mg. per cent, that in the tumour-bearing

FIG. 2.—Relation of tumour size to amino acid nitrogen per cent in blood. Correlation coefficient = 0·7530 ± ·174. Amino acid nitrogen = 0·1213 T.S. +5·63.

mice was 7·40 mg. per cent. It is clear also that this elevation did not take place suddenly, but that it was intimately related to the proportionate size of the tumour, and this association was further demonstrated by the finding of a coefficient of correlation between the two which had the highly significant value of 0·753 ± 0·176. The corresponding regression equation between these variables is: α-amino nitrogen values = 0·1213 tumour percentage size + 5·63. These changes in the level of the blood α-amino nitrogen probably account for the increase in the blood non-protein nitrogen in tumour-bearing animals found by other authors (Greenstein, 1947).

*Blood α-amino nitrogen in mice with regressing tumours.*

In about 10 per cent of the mice used in these experiments the tumour, after having grown and reached a definite size, regressed completely. It seemed of interest to estimate the blood amino acid concentrations in these animals. The findings for 10 such mice are shown in Table III.

TABLE III.—*Blood α-Amino Nitrogen Concentrations in Mice in which a Tumour has Grown and Regressed.*

| Days after inoculation. | Weight of animal (g.). | Blood α-amino nitrogen (mg. per cent). |
|---|---|---|
| 24 | 24 | 4·4 |
| 28 | 31 | 6·3 |
| 36 | 35 | 5·7 |
| 45 | 30 | 4·8 |
| 45 | 28 | 4·2 |
| 45 | 32 | 5·0 |
| 48 | 35 | 4·8 |
| 55 | 40 | 5·0 |
| 60 | 42 | 4·1 |
| 60 | 37 | 4·3 |
| | Mean | 4·86 |

After regression of the tumour, the α-amino nitrogen level of the blood returns almost exactly to its former normal value. A similar tendency to return was observed in 2 mice whose superficial, but growing, tumours were successfully enucleated surgically. The values of their blood α-amino nitrogen five days subsequently were 6·0 and 5·9 mg. per cent.

*The effect of protein-deficient diets on the blood α-amino nitrogen in tumour-bearing mice.*

A study was made of the changes in the blood α-amino nitrogen concentrations in normal and tumour-bearing mice when both had been placed for short periods on a diet that was deficient in protein. The composition of this diet has already been given. The animals consumed this food freely for the first two days, but seemed to lose appetite during the remainder of the experimental five-day period. The loss of weight, which had become severe by the fifth day, together with the blood α-amino nitrogen values, are shown in Table IV.

With the introduction of the deficient diet there was a marked loss of weight in both groups of mice ; the tumours, however, continued to increase in size even at the expense of the host, which was clearly in negative nitrogen balance. The tissues of the host presumably break down to supply the amino-acid needs of the growing parasitic tumour. The early fall in the blood α-amino nitrogen, before any marked loss of weight occurred, seems to indicate the extent to which the concentration of these substances in the blood is dependent upon the state of alimentation. In the normal mice the persistence of the α-amino nitrogen level at about 2 mg. per cent throughout most of the experiment seems to show that this represents the basal level for this group of substances, below which further fall is resisted. In the tumour-bearing group, on the other hand, the concentration of the α-amino nitrogen fell rapidly to between 4 and 5 mg. per cent, and continued at this level for the subsequent three days. The similarity of the fall to that found for the control group would seem to show that whatever metabolic change was responsible for the characteristically elevated blood α-amino nitrogen level was independent of the state of alimentation.

TABLE IV.—*Loss of Weight and Blood α-Amino Nitrogen Concentrations in Control and Tumour-bearing Mice Maintained for Varying Periods on Protein Deficient Diets.*

| Original weight (g.). | Loss of weight (g.). | Duration of diet (days). | Blood α-amino nitrogen (mg. per cent). |
|---|---|---|---|
| | *Controls.* | | |
| 30·1 | 1·2 | 2 | 2·1 |
| 26·5 | 1·5 | 2 | 1·8 |
| 27·4 | 1·6 | 2 | 2·2 |
| 29·5 | 3·7 | 4 | 2·0 |
| 23·5 | 4·1 | 4 | 1·7 |
| 24·6 | 5·1 | 5 | 2·2 |
| 25·5 | 5·6 | 5 | 1·9 |
| 30·8 | 5·4 | 5 | 2·1 |
| | *Tumour-bearing.* | | |
| 25·8 | 1·4 | 2 | 4·8 |
| 25·5 | 1·2 | 2 | 5·0 |
| 28·4 | 2·3 | 2 | 4·8 |
| 29·2 | 4·4 | 4 | 4·7 |
| 27·4 | 5·9 | 4 | 4·0 |
| 30·6 | 6·1 | 5 | 5·1 |
| 31·8 | 6·8 | 5 | 4·7 |
| 32·4 | 4·9 | 5 | 5·4 |

When the surviving mice from each batch were replaced upon the normal diet they put on weight, and the concentration of their blood α-amino nitrogen soon reverted to the earlier levels. Since the protein-deficient diet resulted in equal falls in both groups of mice, the source of the α-amino nitrogen which contributes to the high concentration of these substances in the blood of tumour-bearing animals would appear to be the tumour itself.

### DISCUSSION.

The method of analysis used for this work records specifically all the α-amino groups present in free amino acids, together with those that occur as terminal groups in the non-heat-precipitable polypeptides. The material rise in the values found for these groups in the blood of tumour-bearing mice, roughly about 3 mg. per cent, may thus be in either non-protein nitrogen fraction. There is little evidence that the free α-amino acids are increased in tumorous animals, but observations on rats with a transplantable hepatoma have shown that the second fraction may on average be raised seven or eight fold (Winzler and Burk, 1944). As with the findings here, the raised value reverts to its former normal level should the tumour regress or be resected surgically. It seems probable, therefore, that the rise in the α-amino nitrogen in the blood of our tumour-bearing mice is mostly, if not wholly, attributable to the presence of these polypeptide components.

Normally, the amino acids of the blood are derived from two sources : (i) those that are absorbed from the intestines and have escaped deamination or synthesis into protein in the liver ; and (ii) those liberated by the breakdown of tissues and not yet metabolized. Although it is known that the concentration of amino acids in the blood is raised during the digestion of protein (Bolton and Wright, 1937), the elevation found here in the tumour-bearing animals clearly cannot be accounted for in this way. The elevation continues almost unabated when the animals are placed on a diet wholly deficient in protein. It seems, therefore, that the increase in α-amino nitrogen must be attributable to one or other of the following factors : a local increase in the production of amino acids or of polypeptides by the tumour itself, either through the degeneration of its cells or the hydrolysis of normal body proteins brought to it by the circulation ; or to some hepatic dysfunction that is present in tumour-bearing animals which interferes with the normal disposal of these substances, and thus permits them to accumulate in the blood. This latter possibility will be considered first.

Fujiwara (1929), Weil (1935), Greenstein, Jenrette, Mider and White (1941), and El Mehairy (1949) found little inhibition of liver arginase in tumorous animals. Greenstein (1947) also noted no dimunition in cystine desulphurase activity in the same organ. Arginase and cystine desulphurase are two of the main liver enzymes concerned with the intermediary metabolism of amino acids, and the study of their behaviour provides valuable information upon disturbances in the hepatic enzyme pattern generally. Robertson and Kahler (1942) and Lan (1944) have also failed to observe any decrease in the enzymic activity of livers in animals bearing tumours. From this accumulated evidence the conclusion that the enzymes concerned with the intermediary metabolism of amino acids in the liver are only slightly, if at all, depressed by the growth of the tumour seems justified, provided that this organ is not the seat of primary or secondary neoplasia. Such small depression of liver function as some authors have reported seems insufficient to account for the rise in the values of α-amino nitrogen by over 50 per cent. This conclusion as to hepatic enzymes was supported by the results of histological study of livers from tumour-bearing mice, for numerous sections from such livers revealed no sign of abnormality.

The experiments in which mice were maintained for several days on a diet deficient in nitrogen showed clearly that such treatment brought the blood α-amino nitrogen down to a minimum value below which no further fall took place. In the tumour-bearing group of mice in this experiment the concentration of α-amino nitrogen in the blood was consistently higher than in the control animals ; this difference was almost constant for the first days of the deficient diet to the end of that experimental period. This elevation in the tumour-bearing animals would thus seem to be attributable to the participation of the tumour cells themselves.

Further evidence as to the source of the extra α-amino nitrogen in tumour-bearing mice came from the study of animals with regressing tumours. With the complete disappearance of the tumour the level fell back to within the normal range. That this regression was responsible for the decline in the blood α-amino nitrogen level is corroborated by the finding of a similar reversion in the 2 mice in which progressively growing tumours were resected surgically. It seems, therefore, highly probable that the increase in the concentration of the α-amino nitrogen in the blood is due to the release of peptides or α-amino acids from the tumour itself, possibly as a result of autolysis of dead cells. Such breakdown products probably form in the interior of the tumour mass, where degeneration and necrosis are known to proceed, and leaking thence into the surrounding blood and lymph vessels, gain access to the general circulation.

SUMMARY.

The concentration of α-amino nitrogen in the blood of tumour-bearing mice was found to increase much above that found in comparable normal animals. This increase was correlated with the proportionate size of the tumour. Tumours of less than 5 per cent of the total body weight were not accompanied by any significant change in the α-amino nitrogen concentration in the blood.

The tumour tissue itself is believed to be the source of extra α-amino nitrogen in the blood.

I wish to record my indebtedness to Professor G. Payling Wright for his advice and criticism. My thanks are also due to Dr. R. W. R. Baker for his help and the use of his apparatus.

REFERENCES.

Baker, R. W. R.—(1948) Personal communication.
Becher, E., and Herrmann, E.—(1932) *Desch. Arch. klin. Med.*, **173**, 1.
Bolton, C., and Wright, G. P.—(1937) *J. Physiol.*, **89**, 269.
El Mehairy, M. M.—(1949) Ph.D. Thesis, University of London.
Fujiwara, H.—(1929) *Z. physiol. Chem.*, **185**, 1.
Goldfeder, A.—(1934) *Z. Krebsforsch.*, **40**, 394.
Greenstein, J. P., Jenrette, W. V., Mider, G. B., and White, J.—(1941) *J. nat. Cancer Inst.*, **1**, 687.
*Idem.*—(1947) ' Biochemistry of Cancer.' New York (Academic Press). (Table XXXIV.)
Lan, T. H.—(1944) *Cancer Res.*, **4**, 37.
Malowan, S. L.—(1932) *Schweiz. Arch. Tierheilk.*, **65**, 719.
Robertson, W. V., and Kahler, H.—(1942) *J. nat. Cancer Inst.*, **2**, 595.
Van Slyke, D. D., MacFadyen, D. A., and Hamilton, P.—(1941) *J. biol. Chem.*, **141**, 671.
Weill, L.—(1935) *Ibid.*, **110**, 201.
Winzer, R. J., and Burk, D.—(1944) *J. nat. Cancer Inst.*, **4**, 417.